# The landscape of genetic diseases in Saudi Arabia based on the first 1000 diagnostic panels and exomes

**DOI:** 10.1007/s00439-017-1821-8

**Published:** 2017-06-09

**Authors:** Dorota Monies, Mohamed Abouelhoda, Moeenaldeen AlSayed, Zuhair Alhassnan, Maha Alotaibi, Husam Kayyali, Mohammed Al-Owain, Ayaz Shah, Zuhair Rahbeeni, Mohammad A. Al-Muhaizea, Hamad I. Alzaidan, Edward Cupler, Saeed Bohlega, Eissa Faqeih, Maha Faden, Banan Alyounes, Dyala Jaroudi, Ewa Goljan, Hadeel Elbardisy, Asma Akilan, Renad Albar, Hesham Aldhalaan, Shamshad Gulab, Aziza Chedrawi, Bandar K Al Saud, Wesam Kurdi, Nawal Makhseed, Tahani Alqasim, Heba Y. El Khashab, Hamoud Al-Mousa, Amal Alhashem, Imaduddin Kanaan, Talal Algoufi, Khalid Alsaleem, Talal A. Basha, Fathiya Al-Murshedi, Sameena Khan, Adila Al-Kindy, Maha Alnemer, Sami Al-Hajjar, Suad Alyamani, Hasan Aldhekri, Ali Al-Mehaidib, Rand Arnaout, Omar Dabbagh, Mohammad Shagrani, Dieter Broering, Maha Tulbah, Amal Alqassmi, Maisoon Almugbel, Mohammed AlQuaiz, Abdulaziz Alsaman, Khalid Al-Thihli, Raashda A. Sulaiman, Wajeeh Al-Dekhail, Abeer Alsaegh, Fahad A. Bashiri, Alya Qari, Suzan Alhomadi, Hisham Alkuraya, Mohammed Alsebayel, Muddathir H Hamad, Laszlo Szonyi, Faisal Abaalkhail, Sulaiman M. Al-Mayouf, Hamad Almojalli, Khalid S. Alqadi, Hussien Elsiesy, Taghreed M. Shuaib, Mohammed Zain Seidahmed, Ibraheem Abosoudah, Hana Akleh, Abdulaziz AlGhonaium, Turki M. Alkharfy, Fuad Al Mutairi, Wafa Eyaid, Abdullah Alshanbary, Farrukh R. Sheikh, Fahad I. Alsohaibani, Abdullah Alsonbul, Saeed Al Tala, Soher Balkhy, Randa Bassiouni, Ahmed S. Alenizi, Maged H. Hussein, Saeed Hassan, Mohamed Khalil, Brahim Tabarki, Saad Alshahwan, Amira Oshi, Yasser Sabr, Saad Alsaadoun, Mustafa A. Salih, Sarar Mohamed, Habiba Sultana, Abdullah Tamim, Moayad El-Haj, Saif Alshahrani, Dalal K. Bubshait, Majid Alfadhel, Tariq Faquih, Mohamed El-Kalioby, Shazia Subhani, Zeeshan Shah, Nabil Moghrabi, Brian F. Meyer, Fowzan S. Alkuraya

**Affiliations:** 1 Deparment of Genetics, King Faisal Specialist Hospital and Research Center, Riyadh, Saudi Arabia; 2 Department of Medical Genetics, King Faisal Specialist Hospital and Research Center, Riyadh, Saudi Arabia; 3 Children’s Hospital, King Saud Medical City, Riyadh, Saudi Arabia; 4 Department of Neurosciences, King Faisal Specialist Hospital and Research Center, Jeddah, Saudi Arabia; 5 Department of Pediatrics, Children’s Hospital, Ain Shams University, Cairo, Egypt; 6 Medical Genetic Division, Department of Pediatrics, King Abdullah International Medical Research Centre, King Saud bin Abdulaziz University for Health Sciences, King Abdulaziz Medical City, Riyadh, Saudi Arabia; 7 Department of Pediatrics, King Faisal Specialist Hospital and Research Center, Riyadh, Saudi Arabia; 8 Pediatric Department, Al-Jahra Hospital, Ministry of Health, Kuwait, Kuwait; 9 Department of Obstetrics and Gynecology, King Faisal Specialist Hospital and Research Center, Riyadh, Saudi Arabia; 10 Clinical Genetic Division of Human Genetics & Genome Research, National Research Center, Dokki, Giza, Egypt; 11 Taif Children’s Hospital, Taif, Saudi Arabia; 12 Department of Neurosciences, King Faisal Specialist Hospital and Research Center, Riyadh, Saudi Arabia; 13 Department of Pediatrics, College of Medicine, King Saud University, Riyadh, Saudi Arabia; 14 Department of Pediatrics, Security Forces Hospital, Riyadh, Saudi Arabia; 15 Department of Medicine, King Faisal Specialist Hospital and Research Center, Riyadh, Saudi Arabia; 16 College of Medicine, Alfaisal University, Riyadh, Saudi Arabia; 17 Genetics Department, Sultan Qaboos University Hospital, Muscat, Oman; 18 Department of Pediatrics, Prince Sultan Military Medical City, Riyadh, Saudi Arabia; 19 Department of Liver Transplantation, King Faisal Specialist Hospital and Research Center, Riyadh, Saudi Arabia; 20 Medical Diagnostic Laboratory, Riyadh, Saudi Arabia; 21 Department of Pediatrics, Dr. Suliman Al Habib Medical Group, Riyadh, Saudi Arabia; 22 Saudi German Hospital, Aseer, Saudi Arabia; 23 Department of Pediatrics, King Fahd Hospital of the University, College of Medicine, University of Dammam, Dammam, Saudi Arabia; 24 Department of Pediatric Subspecialties, Children’s Hospital, King Fahad Medical City, Riyadh, Saudi Arabia; 25 Neonatology, Al Hammadi Hospital, Riyadh, Saudi Arabia; 26 Department of Pediatrics, King Faisal Specialist Hospital and Research Center, Jeddah, Saudi Arabia; 27 Pediatric Neurology Department, National Neuroscience Institute, King Fahad Medical City, Riyadh, Saudi Arabia; 28 Pediatric Department, King Abdulaziz University, Jeddah, Saudi Arabia; 29 Global Eye Care, Specialized Medical Center Hospital, Riyadh, Saudi Arabia; 30 Saudi Human Genome Program, King Abdulaziz City for Science and Technology, Riyadh, Saudi Arabia; 31 Maternity and Children’s Hospital, Mecca, Saudi Arabia; 32 Department of Oncology, King Faisal Specialist Hospital and Research Center, Jeddah, Saudi Arabia; 33 Department of Obstetrics and Gynaecology, College of Medicine, King Saud University, Riyadh, Saudi Arabia

## Abstract

In this study, we report the experience of the only reference clinical next-generation sequencing lab in Saudi Arabia with the first 1000 families who span a wide-range of suspected Mendelian phenotypes. A total of 1019 tests were performed in the period of March 2016–December 2016 comprising 972 solo (index only), 14 duo (parents or affected siblings only), and 33 trio (index and parents). Multigene panels accounted for 672 tests, while whole exome sequencing (WES) represented the remaining 347 tests. Pathogenic or likely pathogenic variants that explain the clinical indications were identified in 34% (27% in panels and 43% in exomes), spanning 279 genes and including 165 novel variants. While recessive mutations dominated the landscape of solved cases (71% of mutations, and 97% of which are homozygous), a substantial minority (27%) were solved on the basis of dominant mutations. The highly consanguineous nature of the study population also facilitated homozygosity for many private mutations (only 32.5% of the recessive mutations are founder), as well as the first instances of recessive inheritance of previously assumed strictly dominant disorders (involving *ITPR1*, *VAMP1*, *MCTP2*, and *TBP*). Surprisingly, however, dual molecular diagnosis was only observed in 1.5% of cases. Finally, we have encountered candidate variants in 75 genes (*ABHD6, ACY3, ADGRB2, ADGRG7, AGTPBP1, AHNAK2, AKAP6, ASB3, ATXN1L, C17orf62, CABP1, CCDC186, CCP110, CLSTN2, CNTN3, CNTN5, CTNNA2, CWC22, DMAP1, DMKN, DMXL1, DSCAM, DVL2, ECI1, EP400, EPB41L5, FBXL22, GAP43, GEMIN7, GIT1, GRIK4, GRSF1, GTRP1, HID1, IFNL1, KCNC4, LRRC52, MAP7D3, MCTP2, MED26, MPP7, MRPS35, MTDH, MTMR9, NECAP2, NPAT, NRAP, PAX7, PCNX, PLCH2, PLEKHF1, PTPN12, QKI, RILPL2, RIMKLA, RIMS2, RNF213, ROBO1, SEC16A, SIAH1, SIRT2, SLAIN2, SLC22A20, SMDT1, SRRT, SSTR1, ST20, SYT9, TSPAN6, UBR4, VAMP4, VPS36, WDR59, WDYHV1,* and *WHSC1*) not previously linked to human phenotypes and these are presented to accelerate post-publication matchmaking. Two of these genes were independently mutated in more than one family with similar phenotypes, which substantiates their link to human disease (*AKAP6* in intellectual disability and *UBR4* in early dementia). If the novel candidate disease genes in this cohort are independently confirmed, the yield of WES will have increased to 83%, which suggests that most “negative” clinical exome tests are unsolved due to interpretation rather than technical limitations.

**Electronic supplementary material:**

The online version of this article (doi:10.1007/s00439-017-1821-8) contains supplementary material, which is available to authorized users.

## Introduction

Next generation sequencing (NGS) has ushered in a new era in the delivery of genomic medicine where the genetic diagnosis is no longer limited by the a priori knowledge of the caring physician (Bamshad et al. 2011). The capacity to screen all or a large subset of genes based on broad clinical categories, e.g., developmental delay, rather than a nuanced clinical phenotype, e.g., Malpuech syndrome, has also greatly expanded our knowledge of the relationship between known or putative disease genes and the resulting phenotype (Yang et al. 2013). Indeed, reverse phenotyping is now commonplace in the era of clinical genomics (Alkuraya 2015). The ultimate benefit of improving patient care in terms of precise diagnosis, management and treatment has propelled NGS-based assays to the forefront of modern molecular diagnostics.

Much has been learned from large scale NGS studies in the diagnostic setting. They revealed a higher diagnostic yield (usually around 30%) across clinical indications compared to standard approaches, expanded the genetic and allelic heterogeneity of many conditions, and challenged the concept of “atypical” presentations as instances of dual (or sometimes triple) molecular diagnoses in some patients (Posey et al. 2017; Trujillano et al. 2017). However, very few studies focused on highly consanguineous populations, which are known to differ from outbred populations in a number of ways that impact the landscape of disease-causing mutations (Alfares et al. 2017; Yavarna et al. 2015). For example, we have previously shown that the highly consanguineous nature of the Saudi population is biased in the occurrence of recessive, typically homozygous, mutations in diseases that are typically caused by de novo dominant mutations, e.g., severe intellectual disability (Anazi et al. 2016). These characteristics are predicted to increase the sensitivity of next-generation sequencing-based tests, which has indeed been observed in a few studies (Alfares et al. 2017; Yavarna et al. 2015).

In March 2016, we launched the first reference lab for NGS-based assays in Saudi Arabia. The tests offered include previously validated multigene panels, as well as standard whole exome sequencing (WES) (Group 2015). The high demand for these tests by clinicians from various medical and surgical specialties across the country represented a unique opportunity to observe the distribution of disease-causing mutations in the Saudi population. This is the largest study to date on the mutational spectrum of genetic diseases in the Saudi population in the diagnostic setting. The unselected nature of tested families and their representation of all regions of the Kingdom, allowed us to infer important patterns of genetic diseases in our highly consanguineous population that are relevant to the wider community of diagnostic NGS labs around the world.

## Materials and methods

### Human subjects

All patients underwent testing on a clinical basis with the specific test (panel vs. exome) chosen by their treating physician. A standard informed consent was signed by all patients or their guardians to explain the nature of the test and its potential to reveal secondary findings with the option to decline receiving such findings. Phenotypes were collected as entered by the ordering physician on the requisition forms. For solo tests, only the index was sequenced, while parents were also included in trio tests. Couples who presented with a history of prior affected children were offered duo tests if none of the affected children were available for testing. Duo testing was also rarely requested on two affected siblings.

### Panel tests

Seven clinically themed multigene panels were offered (neuro, dysmorphology/skeletal dysplasia, renal, inborn errors of metabolism, vision, primary immunodeficiency, and gastrointestinal). The gene content and validation of these panels were previously described (Group 2015).

### Whole exome sequencing (WES), variant calling, annotation and autozygome determination

WES was performed using an Agilent Sureselect All Exons V5 (50 Mb) capture kit (Agilent Technologies; Santa Clara, CA, USA) for library preparation. Briefly, DNA was sheared mechanically after which targeted fragments were captured by probe hybridization and amplified before sequencing. An Illumina HiSeq 2500 (Illumina Inc; San Diego, CA, USA) was used for paired-end 100nt sequencing. Sequence alignment, indexing of the reference genome (hg19), variant calling and annotation used a pipeline based on BWA, Samtools, GATK (https://software.broadinstitute.org/gatk/) and Annovar, respectively. Essentially, variants were annotated using a combination of public knowledge databases available from the Annovar package and in-house databases which included collections of previously published Saudi disease causing variants. Autozygome definition solely from whole exome sequence data was undertaken as previously described (Carr et al. 2013).

### Variant interpretation

Our previously described in-house variant interpretation pipeline was used (Group 2015). Briefly, each variant was annotated based on 117 tracks that include chromosomal location, zygosity, number of reads for the reference and alternate alleles, quality metrics, presence within autozygome, local and global frequency data, in silico prediction and previous association with diseases based on HGMD and ClinVar (Harrison et al. 2016; Stenson et al. 2017). Our reporting strategy was as follows: we first considered previously reported disease-causing variants and evaluated them for relevance to the patient’s phenotype. If such variants were identified, we independently evaluated them for likely pathogenicity using local and global population frequency data, as well as in silico prediction of pathogenicity. If our evaluation of the variant is in agreement with the previously reported pathogenicity, we reported them as the likely causal variants. If none was identified, we searched for other relevant variants. Again, such novel variants were evaluated for pathogenicity. In the case of apparently loss-of-function variants with compatible population frequency with the disease in question, we reported these as likely causal. However, in the case of missense and in-frame indels, the burden of proof is much higher according to the ACMG guidelines, so these were usually reported as variants of unknown significance (VOUS) and the result was considered ambiguous (Richards et al. 2015). When only one heterozygous candidate variant was identified in a relevant known recessive disease gene, in the absence of a more compelling candidate, we also opted to report this finding as an ambiguous result. When no candidate variants were identified in known disease genes, we considered variants in genes not previously linked to human disease only when biological relevance was suspected based on a number of factors (pathway known to be implicated, relevant animal models or at least relevant expression data). Results involving novel candidate genes were also considered ambiguous. Only results involving pathogenic or likely pathogenic variants in known disease genes that explain the phenotype in the correct zygosity were labeled “positive”. Patients who opted to receive secondary information (virtually all) only received pathogenic or likely pathogenic variants in the ACMG list of 59 genes recommended for reporting (Kalia et al. 2016).

### Sanger confirmation

After Sanger validation of the first 200 variants, we identified a quality metric above which SNVs had a 99.5% probability of being a true positive. All subsequent candidate variants (other than indels) above this value were reported without Sanger validation and with a disclaimer explaining the above. All candidate indels were Sanger validated before reporting regardless of their quality value.

## Results

### High diagnostic yield of panels and WES in Saudi patients

**Fig. 1:**
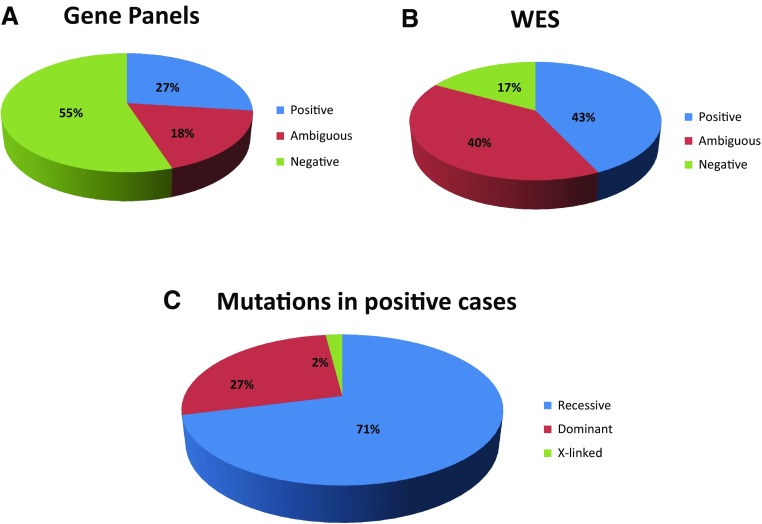
Pie charts showing the yield of the two testing modalities and the breakdown of mutation classes in positive cases

We report in this study the results of the first 1013 families, all indigenous Arabs (originally from Arabia) referred for testing by our diagnostic laboratory (Table S1). One of the seven offered panels was requested in 666 families (645 solo, 16 trio and 5 duo) while WES was requested in the remaining 347 families (321 solo, 17 trio and 9 duo; 15 had WES after receiving negative panel results, i.e., reflex WES) (Table S1). A positive result was reported in 27% of those tested with panels, and 43% with WES (Fig. 1). A breakdown of the diagnostic yield by indication shows marked variability, with the highest yield being multiple congenital malformations in the prenatal setting where the yield was 68%, followed by skeletal dysplasia where the yield was 58% (Table S1). Similarly, duo testing of couples who lost children with a likely genetic diagnosis was associated with a high yield of 50% (83% if novel candidate genes are counted). One notable example is a couple with a history of recurrent non-immune hydrops, one child who died of epidermolysis bullosa and another of osteogenesis imperfecta. Shared carrier status for heterozygous variants in *THSD1*, *ITGB4* and *P3H1*, respectively, was considered relevant (Table S1). Although we always recommended WES when the panel result was negative or ambiguous, reflex WES only accounted for 4% of the WES samples.

### The distribution of disease-mutations in Saudi patients

Autosomal recessive pathogenic and likely pathogenic mutations accounted for the majority of positive cases (235/332, 71%), and were almost always homozygous (97%). This is consistent with the high rate of consanguinity in this cohort. Among the 482 families for whom information on consanguinity was provided, 376 reported consanguinity (78%). In addition, seven families reported endogamy, i.e., intratribal marriage, but no recognizable consanguinity otherwise. Dominant, presumptive *de novo* being the rule, mutations accounted for 27% of positive cases, followed by X-linked mutations (2%) (Fig. 1). In total, pathogenic or likely pathogenic variants spanning 279 known disease genes were identified, 166 of these (43%) are reported here for the first time (Table S1). Of the recessive positive cases, 33% were due to founder mutations (defined as those encountered in other patients or present in the heterozygous state at least once in our in-house database of Saudi variants, which is based on >10,000 Saudi individuals), whereas 67% were private. The frequency of identified pathogenic or likely pathogenic mutations ranged from the most common variant *C12orf57*:NM_001301837:exon1:c.1A>G:p.M1?, representing 1.5% of all positive cases, to the exceedingly rare, e.g., *TRAF3IP2*:NM_001164283:exon4:c.200G>C:p.W67S, which represents only the second mutation in *TRAF3IP2* in the context of severe eczema (Maddirevula et al., in press) (Table S1). We also observed several instances of biallelic pathogenic or likely pathogenic variants in strictly dominant disease genes in the setting of normal parents, e.g., *ITPR1* (Table 1). Dual molecular diagnosis was rare and only accounted for 1.5% of exome-sequenced cases (panel-sequenced cases were not included given the inherent limitation of panel testing to comprehensively detect dual diagnosis) (Table 2). ACMG secondary findings were also very rare and were only identified in 1.2% of exome-sequenced cases. On the other hand, carrier status for previously established Saudi pathogenic mutations was observed in a substantial fraction (90%).

**Table 1:** Recessive mutations in genes only known to cause dominant phenotypes

ID	Variant	Zygosity	Observed phenotype	Reported dominant phenotype
16N-0062	ITPR1:NM_002222:exon50:c.6862C>T:p.R2288X	Hom	GDD and brain atrophy	Spinocerebellar ataxia 15
16W-0091	VAMP1:NM_014231:exon2:c.129 + 1G>A	Hom	Congenital hypotonia, rigid spine, myopathic facies, normal CK	Spastic ataxia 1, autosomal dominant
16W-0191	MCTP2:NM_001159644:exon5:c.384dupT:p.N128 fs	Hom	Severe congenital heart disease, failure to thrive, developmental delay and nephrotic syndrome	Congenital heart disease
16W-0332	TBP:NM_001172085:exon2:c.171delG:p.Q57 fs	Hom	Familial mild ID with difficulty in walking and abnormal movement	Spinocerebellar ataxia 17
16W-0082	VAMP1:NM_001297438:exon2:c.128_129del:p.E43 fs	Hom	Atrial septal defect, fine/gross motor delay, hypotonia, muscle weakness, contractures, pneumonia, congenital myopathy	Spastic ataxia 1, autosomal dominant

**Table 2:** Dual molecular diagnoses

ID	Variant 1	Zygosity	Observed phenotype 1	Variant 2	Zygosity	Observed phenotype 2
16-2491	G6PC:NM_000151:exon2:c.247C>T: p.R83C	Hom	GSD type 1	GAA:NM_000152:exon2:c.266G>A: p.R89H	Hom	GSD type 2
16W-0328	TYRP1:NM_000550:exon8:c.1557T>G: p.Y519X	Het	Abnormal pigmentation	TGIF1:NM_170695:exon1:c.90G>A: p.W30X	Het	Hemimegalencephaly, developmental delay, ADHD
16-2752	RLBP1:NM_000326:exon8:c.773T>G: p.L258 W	Hom	Retinitis pigmentosa	SYT9:NM_175733:exon7:c.1471C>T: p.R491X	Het	Epilepsy
16-2775	LCA5:NM_001122769:exon4:c.763C>T: p.R255X	Hom	LCA	Not identified		Multiple congenital anomalies
16W-0261	CRB1:NM_001193640:exon5:c.1898C>T: p.T633 M	Hom	Retinitis pigmentosa	ADIPOR1:NM_001290553:exon4:c.346C>A:p.P116T	Hom	ID

### Expanding the morbid genome of Mendelian diseases

Pathogenic or likely pathogenic mutations were identified in nine genes with only tentative links to human diseases, thus confirming their classification as bona fide disease genes (Table 3). In addition, our analysis highlighted 75 genes that we propose as novel candidates based on a number of criteria (Table 4). A few such candidates deserve a special emphasis. *AKAP6* was mutated in a patient with intellectual disability (16W-0212). Through our internal matchmaking effort, we were able to identify another de novo truncating variant in the same gene in a patient with intellectual disability: NM_004274.4:c.1572_1573del:p.Lys525Glufs30*. Thus, it seems likely that this is a bona fide disease gene for intellectual disability in humans. Similarly, we have identified independent deleterious variants in *UBR4* in two families with very early onset dementia (16-2737 and 16-2768). This gene is involved in ubiquitin ligation, a mechanism that is impaired in several neurodegenerative diseases so it seems likely that our analysis has uncovered a bone fide autosomal dominant form of early-onset dementia (Parsons et al. 2015). In 16W-0295 we identified an apparently loss-of-function variant in a gene (WHSC1:NM_001042424:exon13:c.2518+1G>A) long suspected to be the candidate gene for manifestations of Wolf-Hirschhorn syndrome, which overlaps significantly with our patient’s features (Nimura et al. 2009).

**Table 3:** Confirming the candidacy of previously reported candidates

ID	Variant	Zygosity	Observed phenotype	Reported tentative link to human disease	Reference
16N-0355	CHRNB2: NM_000748:exon4:c.256-1G>A	Het	Global developmental delay with neuroregression, microcephaly, brain atrophy–temporal lobe atrophy, dystonia, hypertonia, hepatic failure, poor vision	Missense variants in autosomal dominant nocturnal frontal lobe epilepsy	PMID: 7895015
16N-0554	TRAF3IP2:NM_001164283:exon4:c.200G>C:p.W67S	Hom	Severe eczema	Severe eczema	In press
16N-0247	KMT2C:NM_170606:exon36:c.6589C>A:p.Q2197 K	Het	ID	Autism	PMID: 27632392
16N-0255	DENND4B:NM_014856:exon18:c.2702_2703insGCAGC:p.Q901 fs	Het	Cleft palate	Cleft palate	PMID: 25441681
16W-0129	TRMT1:NM_001142554:exon10:c.1245_1246del:p.L415 fs	Hom	ID	ID	PMID: 26308914
16W-0191	MCTP2:NM_001159644:exon5:c.384dupT:p.N128 fs	Hom	Severe congenital heart disease, failure to thrive, developmental delay and nephrotic syndrome	Autosomal dominant coarctation of the aorta	PMID: 23773997
16-2552	ELOVL4:NM_022726:exon5:c.575A>G:p.H192R	Hom	Contractures, ichthyosis, failure to thrive, microcephaly, fine/gross motor delay, speech delay, spasticity, seizures, optic atrophy, prematurity 34 wks, Sjogren Larsson	Ichthyosis and spastic encephalopathy	PMID: 22100072
16-2564	CHD8:NM_001170629:exon26:c.4984C>T:p.R1662X	Het	Fine/gross motor delay, speech delay, learning disability, developmental regression, autistic feature	Autism	PMID: 25961944
16W-0261	ADIPOR1:NM_001290553:exon4:c.346C>A:p.P116T	Hom	ID, microcephaly, global developmental delay, ataxia	ID	PMID: 28097321
16W-0143	CCDC82:NM_024725:exon5:c.904G>T:p.D302Y	Hom	Brain atrophy, suspected white matter disease, ataxia, spasticity, muscle weakness	ID	PMID: 27457812
16-2460	CEP97:NM_001303401:exon11:c.1737G>A:p.W579X	Het	Primordial short stature and hearing deficit, growth parameters below 5th percentile, developmental delay mainly speech delay as well as behavioral disturbances (ADHD), small facial bones, kyphosis in cervical spine and sacrum, coxa valga, and DDH, generalized osteopenia, clinodactyly	ID, solitary kidney, brachydactyly (4th and 5th toes), facial dysmorphism.	PMID: 26539891

**Table 4:** Novel candidates

ID	Variant	Zygosity	Observed phenotype	Justification for candidacy
16-2679	ABHD6:NM_020676:exon3:c.185A>C:p.Y62S	Hom	Fine motor delay, speech delay, intellectual disability/MR, autism spectrum disorder, autistic features, pachygyria, diffuse WM, signal changes, (younger brother)	(a) The nature of the variant (predicted deleterious, novel, within autozygome) and (b) the nature of the gene (involved in synapse function (PMID: 27114538)
16W-0323	ACY3:NM_080658:exon5:c.512C>A:p.A171D	Hom	Microcephaly, seizures, spasticity, brain atrophy	(a) The nature of the variant (novel, predicted deleterious, within autozygome) and (b) the nature of the gene (same family of genes as ACY2 responsible for Canavan disease)
16W-0267	ADGRB2:NM_001294336:exon3:c.21+1G>C	Het	Macrocephaly, fine motor delay, gross motor delay, speech delay, hypotonia	(a) The nature of the variant (novel and potentially truncating) and (b) data showing significant enrichment of ADGRB2 in the brain (PMID: 20367554)
16N-0253	ADGRG7:NM_032787:exon2:c.170C>A:p.T57 N	Hom	Muscle pain following exercise for few years rhabdomyolysis, asymptomatic otherwise, no muscle atrophy or hypertrophy, no tenderness, CK on last visit >15000 following exercise, power is good on examination	(a) The nature of the variant (novel, predicted to be deleterious and within autozygome) and (b) the nature of the gene (this is a G protein-coupled receptor that has been shown to be expressed in skeletal muscle as per ENSBTAG00000012140 in EBI)
16W-0209	AGTPBP1:NM_001286715:exon20:c.2908C > T:p.R970 W	Hom	Failure to thrive, Fine motor delay, gross motor delay, speech delay, intellectual disability/MR, hypotonia, brain atrophy, undescended testis, micropenis, poor vision	(a) The nature of the variant (novel, predicted deleterious, within autozygome) and (b) the nature of the gene (compatible (mouse model PubMed: 11884758)
16W-0085	AHNAK2:NM_138420:exon7:c.6436_6437insGG:p.L2146 fs	Hom	Dysmorphic features, mitral regurgitation, skeletal deformities	(a) The nature of the variant (novel, predicted deleterious, within autozygome) and (b) the nature of the gene (influences FGF1 secretion, which is known to play a role in skeletal development PMID 25560297)
16W-0212	AKAP6:NM_004274:exon4:c.1874A>T:p.Y625F	Het	ID, precocious puberty	(a) The nature of the variant (novel, predicted deleterious) and (b) the nature of the gene (pLI score of 1.00, implicated in cognitive function (PMID: 25644384), the presence of another case with ID and de novo heterozygous truncating variant (see text)
16W-0156	ASB3:NM_001201965:exon5:c.386-3T>C	Hom	Ataxia, Dystonia, Hypertonia, bilateral basal ganglia disease on MRI, brother has epilepsy	(a) The nature of the variant (novel, predicted deleterious, within autozygome) and (b) the nature of the gene (expressed in forebrain PMID: 18817551)
16-2656	ATXN1L:NM_001137675:exon3:c.982C > T:p.R328 W	Het	Ataxia, patient with cerebellar dysfunction beginning since age 5–6 years	(a) The nature of the variant (novel and predicted deleterious) and (b) the nature of the gene (implicated in SCA1 neuropathology PMID 17322884)
16W-0243	C17orf62:NM_001033046:exon3:c.127G>A:p.D43 N	Hom	Growth retardation/short stature, lymphadenopathy, hepatosplenomegaly, recurrent fever, anemia/neutropenia/pancytopenia, HLH rule out	(a) The nature of the variant (novel, predicted deleterious, within autozygome) and (b) the nature of the gene (part of the leukocyte nuclear envelope proteome PMID: 20693407)
16N-0312	CABP1:NM_001033677:exon4:c.922C>T:p.R308X	Het	Microcephaly	(a) The nature of the variant (novel, predicted deleterious) and (b) the nature of the gene (PLI 0.99, encodes a neuronal calcium‐binding protein)
16W-0208	CCDC186:NM_018017:exon2:c.610C>T:p.Q204X	Hom	Failure to thrive, fine motor delay, gross motor delay, speech delay, intellectual disability/MR, hypotonia, brain atrophy, undescended testis, micropenis, poor vision	(a) The nature of the variant (novel, predicted deleterious and within autozygome) and (b) the nature of the gene (encodes a component of dense-core vesicles (DCVs), which are secretory organelles that store and release modulatory neurotransmitters doi: http://dx.doi.org/10.1101/105668)
16N-0321	CCP110: NM_001199022:exon4:c.1355A>G:p.D452G	Het	Tetralogy of Fallot, failure to thrive, growth retardation/short stature, microcephaly, fine motor delay, gross motor delay, speech delay, intellectual disability/MR, hypotonia	(a) The nature of the variant (novel, predicted deleterious) and (b) the nature of the gene (encodes a ciliary protein and its mouse knockout shows overlapping features PMID 26965371)
16W-0333	CLSTN2:NM_022131:exon14:c.2296C > T:p.R766C	Hom	Intellectual disability, autism spectrum disorder, ADHD	(a) The nature of the variant (novel, predicted deleterious), and (b) the nature of the gene (implicated in cognition in mouse PMID: 26171716)
16W-0308	CNTN3:NM_020872:exon17:c.2309C>T:p.P770L	Hom	Microcephaly, intellectual disability/MR, learning disability, severe ADHD, mild motor delay	(a) The nature of the variant (novel and predicted deleterious), and (b) this gene has been found to be expressed in the mouse subventricular zone proliferative cells (PMID: 23914158), which would be consistent with a potential role in the primary microcephaly phenotype this patient has
16W-0185	CNTN5:NM_175566:exon15:c.1955T>G:p.I652R	Het	Fine motor delay, speech delay, intellectual disability/MR, learning disability, developmental regression, hypertonia, seizures tonic, spasticity, cerebellar atrophy, GDD, epilepsy	(a) The nature of the variant (novel and predicted deleterious) and b) the nature of the gene (%HI score of 6 and suggested role in neuronal development PMID: 26391921)
16-2542	CTNNA2:NM_001164883:exon4:c.299G>C:p.G100A	Het	Microcephaly, gross motor delay, speech delay, intellectual disability, learning disability, developmental regression, neuroregression, autistic features	(a) The nature of the variant (novel and predicted deleterious) and b) the nature of the gene (PLI of 1.00 and established role in brain based on the mouse model (PubMed: 9060409)
16W-0318	CWC22:NM_020943:exon20:c.2305_2306del:p.D769 fs	Het	Short stature, and progressive bowing of both legs. DNA analysis for ALPL gen and FGF23 gene are normal (no mutation detected). Skeletal dysplasia of unknown origin	(a) The nature of the variant (novel, predicted deleterious) and (b) the nature of the gene (required for the proper targeting of another skeletal dysplasia gene reported in this paper PMID: 24360810)
16N-0300	DMAP1:NM_019100:exon9:c.1175G>A:p.R392Q	Het	Developmental delay, dysmorphic, hypertelorism, low set ears, ear tag, long philtrum, other extremities	(a) The nature of the variant (novel, predicted deleterious), and (b) the nature of the gene (Tanaka H, Amou R, Shiraki T, Kobayashi M, Nakayama R, Okamoto H. Involvement of DMAP1 in the neural migration and differentiation in zebrafish. Neuroscience Research. 2007 Dec 31;58:S83)
16W-0079	DMKN:NM_001126057:exon5:c.850_851insCA:p.G284 fs	Hom	Ventricular septal defect, speech delay, learning disability, cleft lip/palate, skin laxity, joint laxity	(a) The nature of the variant (novel, truncating, within autozygome) and (b) the nature of the gene (appropriate expression pattern PMID: 17380110)
16-2628	DMXL1:NM_001290321:exon18:c.4256delG:p.C1419fs	Het	Global developmental delay, speech delay, hypotonia, seizure, ptosis, optic disc edema, hypoplastic left heart, congenital heart disease, right UPJ obstruction with grade v hydronephrosis and dysmorphic features. Neuro panel is negative	(a) The nature of the variant (predicted deleterious, novel) and b) the nature of the gene (PLI score of 1.00, its Drosophila ortholog KO has abnormal development PMID: 25259927, and has been proposed to play a role in the pathogenesis of 5q22.3q23.3 microdeletion syndrome PMID: 15742475)
16W-0265	DSCAM:NM_001271534:exon23:c.4132+2T>A	Hom	Growth retardation/short stature, microcephaly, fine motor delay, gross motor delay, speech delay, intellectual disability/MR, seizure	(a) Nature of the variant (novel and potentially truncating), and (b) this gene has an extensively studied role in axon development in the CNS (PubMed: 10892653)
16W-0183	DVL2:NM_004422:exon14:c.1690C > T:p.Q564X	Hom	Cardiomyopathy, short stature, ptosis, facial dysmorphism, developmental delay	(a) The nature of the variant (novel, predicted deleterious, within a run of homozygosity) and (b) the nature of the gene (compatible mouse knockout phenotype PMID: 12421720)
16W-0102	ECI1:NM_001919:exon5:c.563+1G>T	Hom	Fine motor delay, gross motor delay, speech delay, Intellectual disability/MR, learning disability, hypertonia, dysmyelinating disease	(a) The nature of the variant (novel, predicted deleterious, within a run of homozygosity) and (b) the nature of the gene (this gene encodes mitochondrial 3, 2-*trans*-enoyl-CoA isomerase which catalyzes the shift of the double bonds of 3-*cis*- and 3-*trans*-isomers to the 2-*trans*-enoyl-CoAs, which are substrates of the 2-*trans*-enoyl-CoA hydratase)
16N-0280	EP400:NM_015409:exon47:c.8226_8227insGCAACAG:p.Q2742 fs	Het	GDD, epilepsy and CHD	(a) The nature of the variant (novel, truncating) and (b) the nature of the gene (it has a PLI score of 1, and is a chromatin modulator (PMID: 26669263), which is a class commonly mutated in intellectual disability and congenital heart disease)
16W-0213	EPB41L5:NM_001184939:exon14:c.1178+1G>T	Het	Fine motor delay, hypertelorism of eye, panhypopituitarism, myelomeningocele, dislocated hips, scoliosis	(a) The nature of the variant (novel, predicted deleterious) and (b) the nature of the gene (implicated in the specification and differentiation of neurons PMID: 27510968)
16W-0230	FBXL22:NM_203373:exon1:c.279G>C:p.K93 N	Hom	Muscle weakness, proximal lower extremity, calf prominence, CK 5000 increase, LGMD phenotype, DMD (at MDL) negative, NGS (myopathy) at MNG lab negative	(a) The nature of the variant (novel, predicted deleterious, within autozygome) and (b) the nature of the gene (implicated in sarcomere physiology PMID: 22972877)
16-2696	GAP43:NM_002045:exon3:c.629-3C>T	Het	Motor neuron disease, paraparesis, spastic with upper and lower motor neuron signs	(a) The nature of the variant (predicted deleterious, novel) and (b) the nature of the gene (involved in axon regeneration (PubMed: 7859286)
16W-0312	GEMIN7:NM_001007269:exon2:c.154G>A:p.E52 K	Hom	Gross motor delay, speech delay, normal metabolic, normal MRI, hypotonia, abnormal movements, myoclonic but no seizures	(a) The nature of the variant (novel, predicted deleterious, within autozygome), and (b) the nature of the gene (implicated in motor neuron survival PMID: 12065586)
16W-0320	GIT1:NM_001085454:exon5:c.611T>A:p.I204 N		No information	(a) The nature of the variant (novel, predicted deleterious), and (b) the nature of the gene (PLI 1.00, relevant mouse phenotype PMID: 25997734)
16W-0313	GRIK4:NM_001282470:exon19:c.2479T>G:p.F827 V	Hom	Microcephaly, developmental regression, patient niece have severe GDD, patient sister died at 3 years old with GDD	(a) The nature of the variant (novel, predicted deleterious, within autozygome), and (b) the nature of the gene (implicated in autism pathogenesis PMID: 26446216)
16W-0218	GRSF1:NM_001098477:exon3:c.88C>T:p.R30X	Het	Speech delay, intellectual disability/MR, learning disability, developmental regression, seizures- tonic–clonic, refractory epilepsy, neuro regression	(a) The nature of the variant (novel, predicted deleterious) and (b) the nature of the gene (implicated in brain development PMID: 18593884)//Novel candidate
16W-0115	GRTP1:NM_001286732:exon6:c.698G>A:p.W233X	Hom	Unexplained cholestasis with elevated liver enzymes	(a) The nature of the variant (novel, predicted deleterious, within autozygome) and (b) the nature of the gene (encodes a liver-enriched protein PMID: 11564724)
16W-0277	HID1:NM_030630:exon19:c.2318dupC:p.P773 fs	Hom	Failure to thrive, growth retardation/short stature, intellectual disability/MR, agenesis for corpus callosum, hypotonia, panhypopituitarism, similar F/H form sever maternal cousins (males) likely X- linked one bother dies at age of 6 months, 2 sons of his aunt dies with similar problem but no diagnosis, hypoglycemia, central hypothyroidism, central adrenal insufficiency, post meningitis	(a) The nature of the variant (novel, predicted deleterious, within autozygome) and (b) the nature of the gene (required for homotypic fusion of immature secretory granules during maturation PMID: 27751232)
16-2731	IFNL1:NM_172140:exon3:c.305T>C:p.L102P		Failure to thrive, microcephaly, fine motor delay, gross motor delay, speech delay, agenesis of corpus callosum, migration disorder, hypotonia, scoliosis, absent thumb, IUGR, external ear malformation, epilepsy and skeletal abnormalities	(a) The nature of the variant (predicted deleterious, novel, within ROH) and (b) the nature of the gene (involved in DNA damage repair, a mechanism that is impaired in several syndromes with overlapping clinical features (PMID: 25692705)
16W-0222	KCNC4:NM_001039574:exon1:c.23C>T:p.S8F	Het	Ataxia, dystonia	(a) The nature of the variant (novel, predicted deleterious), (b) its known expression pattern in brain and altered expression in patients with neurodegenerative diseases (PMID: 21912965)
16W-0292	LRRC52:NM_001005214:exon2:c.659delT:p.L220 fs	Het	Muscle weakness	(a) The nature of the variant (novel, predicted deleterious) and (b) the nature of the gene (modifies BK potassium channels and is strongly expressed in skeletal muscles PMID: 22547800)
16W-0157	MAP7D3:NM_001173517:exon7:c.804_805insTA:p.D269_A270delinsX	Hemi	ADHD with GDD, no speech, mild hepatosplenomegaly	(a) The nature of the variant (novel and truncating) and (b) the nature of the gene (expressed in brain and suggested to play a role in ID PMID: 24817631)
16W-0191	MCTP2:NM_001159644:exon5:c.384dupT:p.N128 fs	Hom	AV pulmonary AV malformation, Failure to thrive, growth retardation, speech delay, learning disability, hearing loss, nephrotic syndrome	(a) The nature of the variant (novel and predicted deleterious) and (b) the nature of the gene (PLI score of 0.98 and the neurological phenotype in the mouse model PubMed: 22198669)
16W-0176	MED26:NM_004831:exon3:c.1771T>G:p.L591 V	Het	Fine motor delay, speech delay, intellectual disability/MR, stereotypic behaviours	(a) The nature of the variant (novel and predicted deleterious) and (b) the nature of the gene (PLI socre of 0.95 and established role in regulating neuronal gene expression PMID: 19049968)
16W-0204	MPP7:NM_173496:exon16:c.1251C>G:p.Y417X	Hom	Ataxia	(a) The nature of the variant (novel, predicted deleterious and within autozygome) and (b) the nature of the gene (encodes a member of membrane-associated guanylate kinase (MAGUK) family of proteins, which are implicated in synapse formation and function PMID: 21739617)
16W-0288	MRPS35:NM_021821:exon1:c.112+1->T	Hom	Failure to thrive, fine motor delay, gross motor delay, speech delay, intellectual disability/MR, brain atrophy, abnormal shaper of skull and limbs, facial dimorphism.	(a) The nature of the variant (novel, predicted deleterious, within autozygome) and (b) the nature of the gene (the same family of proteins has been implicated in a number of mitochondrial multisystem disorders)
16W-0219	MTDH:NM_178812:exon6:c.862delG:p.E288 fs	Hom	Growth retardation/short stature, fine motor delay, gross motor delay, speech delay, intellectual disability/MR, learning disability, developmental regression, autism spectrum disorder, autistic features, stereotypic behaviours, other psychiatric symptoms	(a) The nature of the variant (homozygous truncation), (b) it is known to be expressed in brain and has been extensively studied in the context of glioma (PMID: 28107197)
16W-0304	MTMR9:NM_015458:exon9:c.1415A>T:p.N472I	Hom	Gross motor delay, speech delay, intellectual disability/MR, seizures, two sisters are similarly affected	(a) The nature of the variant (novel, predicted deleterious, within autozygome) and (b) the nature of the gene (MTMR7 forms a complex with MTMR9 and dephosphorylates phosphatidylinositol 3-phosphate and Ins(1, 3)P2 in neuronal cells PMID: 12890864
16N-0329	NECAP2:NM_001145277:exon3:c.212C>A:p.A71D	Hom	GDD and cataract	(a) The nature of the variant (novel, predicted deleterious, within autozygome) and (b) the nature of the gene (mutations in its paralog NECAP1 cause GDD PMID 24399846)
16-2728	NPAT:NM_002519:exon17:c.3644C>T:p.S1215L	Het	Psychomotor retardation, seizures disorder, Angelman syndrome	(a) The nature of the variant (predicted deleterious, novel) and (b) the nature of the gene (low %HI score in DECIPHER and involved in chromatin remodelling which is a common mechanism in neurodevelopmental disorders
16W-0086	NRAP:NM_001261463:exon5:c.400_407del:p.C134 fs	Hom	Cardiomyopathy (dilated)	(a) The nature of the variant (novel, predicted deleterious, within autozygome) and (b) the nature of the gene (implicated in cardiomyopathy pathogenesis in mouse PMID: 21276443)
16W-0210	PAX7:NM_001135254:exon3:c.433C>T:p.R145X	Het	Hypotonia, exercise intolerance/easy fatigue, muscle weakness, stroke/stroke-like episodes, recurrent headache/migraine, creatine phosphokinase abnormalities	(a) The nature of the variant (novel, predicted deleterious) and (b) the nature of the gene (low %HI of 11.2, established role in skeletal muscle development PMID: 21954137)
16W-0150	PCNX:NM_001308160:exon26:c.4804A>G:p.K1602E	Het	Atrial septal defect, ventricular septal defect, failure to thrive, fine/gross motor retardation, brain atrophy, dandy walker variant, hypotonia, hypertonia, seizures myoclonic, spasticity, prematurity, IUGR, gastrointestinal reflux, 8 months old female with GDD-epilepsy, SHC, abnormal karyotype 46 XX add 3P	(a) The nature of the variant (novel, predicted deleterious) and (b) the nature of the gene (high PLI of 1 and established expression in mouse brain)
16W-0148	PLCH2:NM_001303012:exon16:c.2132G>A:p.C711Y	Hom	Failure to thrive, fine motor delay, gross motor delay, speech delay, autistic features, hypotonia, seizure, white matter changes in bilateral p…, poor vision, GDD and epilepsy	(a) The nature of the variant (novel, predicted deleterious and within ROH) and (b) the nature of the gene [its ortholog PLCH1 mutations causes an overlapping phenotype (PMID: 28413018)]
16W-0083	PLEKHF1:NM_024310:exon2:c.514C>T:p.R172C	Hom	Myopathy, cardiomyopathy (Dilated), muscle weakness	a) The nature of the variant (novel, predicted deleterious, within autozygome) and b) the nature of the gene (highest expression was detected in heart and skeletal muscle)
16N-0289	PTPN12:NM_001131009:exon12:c.904C>T:p.R302X	Het	Multiple epiphyseal dysplasia	(a) The nature of the variant (novel, truncating) and (b) the nature of the gene (it has a PLI score of 1, and is involved in the regulation of multimeric protein complexes in podosomes of osteoclasts PMID 16052478)
16W-0097	QKI:NM_206853:exon6:c.635-2A>T	Het	Dystonia, (the rest is unclear)	(a) The nature of the variant (novel, predicted deleterious) and (b) the nature of the gene (high PLI score of 0.96 and established mouse model that manifests severe neurological disease PubMed: 8589716)
16N-0249	RILPL2:NM_145058:exon1:c.161C>T:p.A54 V	Hom	Skeletal dysplasia, very mild speech delay, gross motor delay, learning disability, facial dysmorphism	(a) The nature of the variant (novel, predicted to be deleterious and within a run of homozygous) and (b) the nature of the gene (this is a candidate ciliopathy gene as per PMID: 23264467 so this may represent a skeletal ciliopathy
16W-0154	RIMKLA:NM_173642:exon1:c.18G>A:p.W6X	Hom	Recurrent chest infection, sickle cell trait, recurrent spells of absent spells, carrier of MEVF gene, IEG negative	(a) The nature of the variant (novel, predicted deleterious, within autozygome) and (b) the nature of the gene (enzyme responsible for making NAAG, an endogenous peptide abundant in mammalian nervous systems PMID: 16127367)
16-2732	RIMS2:NM_014677:exon2:c.418C>T:p.R140C	Het	Lower limb muscle weakness and spasticity, periventricular leukomalacia, normal CK	(a) The nature of the variant (novel and predicted deleterious) and (b) the nature of the gene (PLI of 1.00 and established role in synapse formation PubMed: 12620390)
16W-0075	RNF213:NM_001256071:exon60:c.14394delC:p.F4798 fs	Het	Autism spectrum disorder	(a) The nature of the variant (novel, predicted deleterious) and (b) the nature of the gene (associated with CNS vascular malformation PMID 27745834)
16W-0152	ROBO1:NM_133631:exon21:c.2990A>T:p.D997 V	Het	Intellectual disability, epilepsy, autism, ADHD, mitral regurgitation	(a) The nature of the variant (novel and predicted deleterious) and (b) the nature of the gene (%HI of 3, and suggested link to autism PMID: 18270976)
16-2670	SEC16A:NM_001276418:exon5:c.3820C>T:p.R1274C	Hom	Microcephaly, fine motor delay, gross motor delay, speech delay, abnormal nails—small fingers, flat feet, short stature	(a) The nature of the variant (predicted deleterious, novel, within autozygome) and (b) the nature of the gene (involved in proliferation PMID PMID: 25526736)
16N-0301	SIAH1:NM_001006610:exon1:c.91_91del:p.E31 fs	Hom	Developmental delay, seizure disorder	(a) The nature of the variant (novel, truncating) and (b) the nature of the gene (it functions as an E3 ubiquitin ligase that binds to two presynaptic active zone proteins Piccolo and Bassoon PMID: 28231469)
16-2718	SIRT2:NM_001193286:exon3:c.70G>T:p.E24X	Hom	Microcephaly, fine/gross motor delay, speech delay, learning disability, developmental regression, periventricular leukomalacia, prematurity, oligohydramnios, nystagmus, recurrent fever	(a) The nature of the variant (predicted deleterious, novel, within autozygome) and (b) the nature of the gene (implicated in myelination PMID:21949390)
16W-0147	SLAIN2:NM_020846:exon5:c.938C>T:p.T313I	Het	Microcephaly, learning disability, developmental regression, sever hypotonia, seizures (?infantile spasms), delayed myelination, sever epileptic encephalopathy (1.5)	(a) The nature of the variant (novel, predicted deleterious) and (b) the nature of the gene (high PLI of 0.88 and established role in neuronal development PMID: 23077057)
16W-0319	SLC22A20:NM_001004326:exon4:c.763+1G>A	Hom	Epilepsy, GDD, fine motor delay, gross motor delay, speech delay, intellectual disability learning disability, stereotypic behaviors, dystonia, hypotonia, seizures (GTC), spasticity	(a) The nature of the variant (novel, predicted deleterious, within autozygome) and (b) the nature of the gene (high expression in rat olfactory bulb and forebrain PubMed: 17714910)
16W-0283	SMDT1:NM_033318:exon2:c.255C>G:p.S85R	Hom	LGMD?, dystonia	(a) The nature of the variant (novel, predicted deleterious, within autozygome) and (b) the nature of the gene (encodes EMRE, an essential component of mitochondrial Ca uniporter, which has been implicated in mitochondrial myopathies PMID: 24231807)
16W-0193	SRRT:NM_001128852:exon5:c.497C>T:p.T166 M	Het	Failure to thrive, fine motor delay, gross motor delay, speech delay, intellectual disability	(a) The nature of the variant (novel and predicted deleterious) and (b) the nature of the gene (PLI score of 0.98 and the neurological phenotype in the mouse model PubMed: 22198669)
16-2541	SSTR1:NM_001049:exon3:c.1169_1170insGCTCTGAGCCCGGGCCACGCAGGG:p.T390delinsTLX	Hom	Hypotonia, developmental delay	(a) The nature of the variant (novel, within autozygome and predicted deleterious), and (b) the nature of the gene (this serotonin receptor is strongly expressed in brain and GI tissues, has been linked to neuroendocrine tumors that manifest as unexplained diarrhea and the knockout mouse has hypoactivity (PMID 7777168)
16W-0263	ST20:NM_001100879:exon3:c.135T>A:p.C45X	Het	No information	(a) Nature of the variant (novel and truncating), (b) the gene is known to be a tumor suppressor so it is conceivable that this patient’s germline ST20 variant may have predisposed her to the bilateral optic nerve tumor observed. Confirming LOH in a tumor sample will greatly corroborate this hypothesis and this can be done on research basis (please contact lab director to coordinate)
16-2752	SYT9:NM_175733:exon7:c.1471C>T:p.R491X	Hom	Seizures, retinitis pigmentosa, obesity	(a) The nature of the variant (predicted deleterious, novel) and (b) the nature of the gene (%HI score of 14, and its deficiency in mouse causes severely impaired synaptic transmission (PubMed: 17521570)
16W-0255	TSPAN6: NM_001278740:exon7:c.513+4C>T	Hemi	Fine motor delay, gross motor delay, speech delay, intellectual disability, learning disability, head nodding nystagmus, increase myopia, decrease vision with age, previous neuro-panel negative	(a) The nature of the variant (novel, predicted deleterious, hemizygous) and (b) the nature of the gene (regulates hippocampal synaptic transmission and long term plasticity PMID: 28207852)
16-2737	UBR4:NM_020765:exon22:c.2873C>A:p.S958Y	Het	Developmental regression, brain atrophy, ataxia,	(a) The nature of the variant (predicted deleterious, novel) and (b) the nature of the gene (PLI score of 1.00, involved in ubiquitin ligation, a mechanism that is impaired in several neurodegenerative diseases, the presence of another case with early dementia and mutation in the same gene)
16-2768	UBR4:NM_020765:exon66:c.9787G>A:p.A3263T	Het	Behavioral changes starts 4 year ago, then seizures, finally developed dementia white matter changes.	(a) The nature of the variant (predicted deleterious, novel) and (b) the nature of the gene (PLI score of 1.00, involved in ubiquitin ligation, a mechanism that is impaired in several neurodegenerative diseases, the presence of another case with early dementia and mutation in the same gene)
16W-0282	VAMP4:NM_001185127:exon5:c.175G>A:p.V59 M	Hom	Microcephaly, speech delay, intellectual disability/MR, cataracts	(a) The nature of the variant (novel and predicted deleterious), and (b) the published role of VAMP4 in synaptic transmission (PMID: 22406549)
16W-0233	VPS36:NM_001282169:exon14:c.894-2->T	Hom	Speech delay, intellectual disability/MR	(a) The nature of the variant (novel, predicted deleterious, within autozygome) and (b) the nature of the gene (mutations in other components of ESCRT-II are also known to cause ID such as PMID: 22717650)
16W-0197	WDR59:NM_030581:exon4:c.270G>A:p.W90X	Het	Fine motor delay, gross motor delay, dystonia, hypertonia, seizures: Inf. spasm, spasticity	(a) The nature of the variant (novel, predicted deleterious) and (b) the nature of the gene (encodes a component of GATOR complex, which is implicated in familial focal epilepsies and focal cortical dysplasia PMID 27173016)
16N-0291 (duo)	WDYHV1:NM_001283024:exon5:c.256C>T:p.R86X	Het	Father of an affected child	(a) The nature of the variant (extremely rare, truncating) and (b) the nature of the gene (it encodes NTAQ1, a component of the N-terminal pathway in mammalian cells, which has been implicated in the pathogenesis of neurodegenerative diseases PMID: 23499006)
16W-0295	WHSC1:NM_001042424:exon13:c.2518+1G>A	Het	Failure to thrive, facial dysmorphism, triangular face mild	(a) The nature of the variant (novel, predicted deleterious) and (b) the nature of the gene (pLI 1.00, this gene has long been suspected to be the candidate gene for Wolf-Hirschhorn syndrome, which has significant overlap with the provided features PubMed: 19483677)

### Phenotypic expansion

Table 5 summarizes positive cases in which the molecular lesion was unexpected, given the established phenotype for the respective gene. For example, 16N-0149 presented with a CHARGE-like presentation but was found to have a de novo variant in *KMT2A*, the gene responsible for Kabuki syndrome. We have previously suggested the overlap between CHARGE and Kabuki to be biologically relevant and this has been confirmed very recently by a large study (Butcher et al. 2017; Patel and Alkuraya 2015). Similarly, 16W-0253 presented with progressive spasticity, hyper-reflexia and behavioral changes due to a homozygous truncating mutation in *FAM134B*. This presentation is different from what has been different in the context of *FAM134B* mutation, which is a form of hereditary sensory and autonomic neuropathy characterized by early childhood onset of distal sensory impairment usually resulting in ulceration and associated with variable autonomic features, such as hyperhidrosis and urinary incontinence (Ilgaz Aydinlar et al. 2014; Kurth et al. 2009).

**Table 5:** Atypical phenotypes

ID	Variant	Zygosity	Observed phenotype	Reported phenotype
16N-0062	ITPR1:NM_002222:exon50:c.6862C>T:p.R2288X	Hom	GDD and brain atrophy	Spinocerebellar ataxia 15
16N-0149	KMT2A:NM_001197104:exon4:c.3248G>A:p.R1083Q	Het	CHARGE-like	Kabuki syndrome
16N-0235	WNT10B:NM_003394:exon4:c.338-1G>C	Hom	Split foot malformation and hand polydactyly	Split-hand/foot malformation 6
16 N-0395	SMPD1:NM_000543:exon4:c.1267C>T:p.H423Y	Hom	Severe neonatal cholestasis	Niemann-pick disease
16N-0439	RAD21:NM_006265:exon2:c.68G>A:p.W23X	Het	Overgrowth, macrocephaly, ID, ataxia, renal malformation, renal tubulopathy, ptosis, anorectal malformation, AML	Cornelia de lange syndrome
16N-0481	HSD3B7:NM_025193:exon6:c.694+2T>-	Hom	High GGT cholestasis in infancy that later resolved with normal GGT and serum bilirubin but with elevated liver enzymes suggestive of chronic compensated liver disease, liver biopsy suggested PFIC type 3	Bile acid synthesis defect, congenital, 1
16 N-0617	PRKCSH:NM_001289103:exon17:c.1462-1G>C	Het	Idiopathic chronic liver disease, no cysts	Polycystic liver disease
16N-0653	ITGB2:NM_000211:exon13:c.1756C>T:p.R586 W	Hom	Inflammatory bowel disease	Leukocyte adhesion deficiency
16N-0703	PKD2:NM_000297:exon1:c.567G>A:p.W189X	Het	Nephrolithiasis in the 2nd yr of life	Autosomal dominant polycystic kidney disease
16-2567	COG6: NM_020751.2:c.1167-24A>G	Hom	Failure to thrive, global developmental delay, hiatus hernia, lung hypoplasia, congenital diaphragmatic hernia, low intestinal motility disorder, partial intestinal obstruction	ID and anhidrosis
16W-0091	VAMP1:NM_014231:exon2:c.129+1G>A	Hom	Congenital hypotonia, rigid spine, myopathic facies, normal CK	Spastic ataxia 1, autosomal dominant
16W-0235	RFT1: NM_052859:exon7:c.775+1G>C	Hom	Congenital microcephaly, GDD, epilepsy, hematemesis	marked developmental delay, hypotonia, seizures, hepatomegaly, and coagulopathy
16W-0253	FAM134B:NM_019000:exon6:c.503C>G:p.S168X	Hom	Behavioral changes, muscle weakness, frequent falls, brisk reflexes, suspected HSP	Neuropathy, hereditary sensory and autonomic, type IIB
16 W-0291	PHKA1:NM_001122670:exon12:c.1174C>T:p.R392X	Hom	Dystonic posturing of the upper limbs, spasticity of the lower limbs, FTT, learning disability	Muscle glycogenosis
16W-0321	PIEZO2:NM_022068:exon3:c.273_279del:p.A91 fs	Hom	Severe lower limb weakness, severe progressive scoliosis, suspected SMA, no arthrogryposis	Arthrogryposis
16W-0328	TGIF1:NM_170695:exon1:c.90G>A:p.W30X	Het	Hemimegalencephaly, developmental delay, ADHD, Abnormal pigmentation, all over the body, normal karyotype	Holoprosencephaly 4
16N-0505	DCC:NM_005215:exon9:c.1423C>T:p.R475X	Het	Adult onset ataxia slow progressive. No cognitive dysfunction. NCS: pred. sensory neuropathy	Mirror movements 1

### Impact on management

With very few exceptions, solved cases were positive for genes not suspected a priori by the ordering physician according to the information provided. Furthermore, the correct molecular characterization of many patients led to immediate implementation of management changes. Examples include a child with maple syrup urine disease who was missed by newborn screening and presented at 6 m of age with failure to thrive, developmental regression, abnormal hair, exfoliative skin lesions and epilepsy (16N-0566). 16N-0616 presented with intellectual disability, which was misdiagnosed as hypoxic ischemic encephalopathy but was found to have a novel startloss *PTEN* mutation, thus enabling the treating physician to implement active tumor surveillance. 16N-0617 is 2-year-old child who presented with unexplained chronic liver failure with hyperbilirubinemia and was being considered for liver transplant. The finding that he has polycystic liver disease (no cysts were identified on ultrasonography) caused by an autosomal dominant PRKCSH:NM_001289103:exon17:c.1462-1G>C mutation was very helpful in selecting an unaffected donor from his relatives. 16N-0653 was managed for many years with anti-inflammatory agents to treat her “Crohn’s disease” but she was found to have leukocyte adhesion molecule deficiency caused by ITGB2:NM_000211:exon13:c.1756C>T:p.R586 W resulting in a drastically different management. 16N-0703 presented in the 2nd year of life with severe nephrolithiasis and his management was significantly altered when he was found to have autosomal dominant polycystic kidney disease (PKD2:NM_000297:exon1:c.567G>A:p.W189X), a known risk factor of nephrolithiasis although his very early presentation was unusual (Grampsas et al. 2000). 16W-0247 is an 8-year-old child who was followed for seizures, ataxia and learning disability but the diagnosis of neurofibromatosis type I was missed until exome sequencing revealed a previously known pathogenic mutation in *NF1*. 16W-0269 is a 6-year-old child who was managed for years as a case of Bartter syndrome but exome sequencing led to a change in management after revealing a pathogenic mutation in *SLC26A3* thus establishing the correct diagnosis of chloride-losing diarrhea. 16-2717 is a 2-year-old child whose diagnosis of pyridoxine-dependent epilepsy was missed until revealed by exome sequencing leading to immediate initiation of pyridoxine.

## Discussion

Genetic studies on the Saudi population have contributed significantly to the global effort of identifying the causal mutations of Mendelian diseases, particularly in the area of autosomal recessive diseases (Alkuraya 2014). The very characteristics that make this population a significant contributor to the analysis of recessive diseases, i.e., consanguinity and large family size, have also recently been exploited to advance our knowledge of other areas of human genetics research, including dominant disorders (by reclassifying dominant mutations when observed in homozygosity in individuals who lack the reported dominant phenotype) and even common diseases (by identifying Mendelian phenocopies and by challenging or supporting GWAS candidate genes when observed in individuals who are functional “knockouts”, i.e., homozygous for loss-of-function alleles as a function of autozygosity) (Abouelhoda et al. 2016; Monies et al. 2017) (Maddirevula et al., in press). However, despite the wealth of published data on genetic diseases in Saudi Arabia, unbiased data on the overall distribution of these diseases and their genetic landscape are largely lacking.

Our molecular diagnostic lab is the sole major referral NGS laboratory in Saudi Arabia. As such, it receives samples from patients with suspected genetic disorders from all healthcare sectors in the country (private hospitals and practices, as well as state-funded healthcare system). This provided us with a unique opportunity to observe and report a wide spectrum of phenotypes from all regions of the country, as well as other countries in Arabia (Oman and Kuwait) in a largely unbiased fashion. In this report, we describe our laboratory’s prospective analysis of families referred for panel or exome sequencing. The unbiased selection of the first 1000 samples has allowed us to derive important conclusions about the nature of genetic diseases in the country.

Autosomal recessive disorders, as expected, accounted for the bulk of Mendelian disease burden. This is consistent with several previous reports, including the very recent report by Alfares and colleagues who retrospectively analyzed 454 families that underwent exome sequencing by various commercial labs although they did not provide phenotyping information (Alfares et al. 2017). It is noteworthy that enhanced autozygosity also led to several instances of autosomal recessive inheritance of strictly dominant genes. These cases can improve our understanding of the molecular pathogenesis of the dominant counterpart (Monies et al. 2017). For example, we report two families with a severe congenital myopathy phenotype each with a different homozygous truncating mutation in *VAMP1* inherited from healthy non-ataxic parents. This suggests that the dominant spinocerebellar ataxia previously reported in a family with a heterozygous *VAMP1* mutation may not be caused by haploinsufficiency as originally suggested but a different mechanism, e.g., dominant negative (Bourassa et al. 2012). Furthermore, the difference in phenotype between the observed recessive and previously reported dominant phenotypes should serve as a reminder that caution is warranted before dismissing variants in genes deemed “irrelevant” to the phenotype in question. These cases also provide an alternative explanation to non-manifesting carriers of clearly pathogenic mutations in dominant genes other than reduced penetrance, i.e., truly recessive inheritance. The predominance of autosomal recessive disorders also allowed us to perform “molecular autopsy by proxy” in families who lost children in the past due to recessive conditions but have no material left to test those children directly. Duo analysis of the parents allowed us to identify the likely causal mutation in the overwhelming majority. The highlighted couple who lost three pregnancies/neonates with three different disorders all of which were identified by duo exome sequencing is an example of the power of this approach. It may be surprising that despite this trend, we have only confirmed dual molecular diagnosis in 1.5% of our cohort. This could be attributed to our methodology (we only considered dual diagnosis if both molecular lesions could be classified as at least likely pathogenic).

Secondary findings represent a complicated ethical and practical issue in genomic sequencing. In our analysis, we followed the ACMG recommendations of reporting only pathogenic/likely pathogenic variants in the list of 59 actionable genes (Kalia et al. 2016). Only 1.2% of our cohort received positive results for this class of secondary findings, consistent with previous studies (Dheensa et al. 2016). However, we also opted to report carrier status for a select list of pathogenic recessive mutations that we have previously characterized in our population. Although it is generally discouraged to disclose these results in the pediatric setting, the very high rate of consanguinity in our population prompted us to consider the consequences of not disclosing this information. The carrier status of the index essentially indicates that at least one parent is a carrier, so if the other parent is related then the probability of both parents being carriers of the same mutant allele may not be rare. Thus, we considered this an opportunity to prevent the occurrence of another recessive disease unrelated to the disease being tested by counseling parents about this possibility and offering them the option of being tested for their carrier status. This class of secondary findings was positive in a large fraction of WES cases (90%).

The establishment of novel links between genes and diseases remains a priority in human genetics research to enable the molecular diagnosis of all Mendelian diseases in the near future. The final percentage of genes that are linked to Mendelian phenotypes remains a highly contested speculation (Boycott et al. 2017). However, large iterative sequencing efforts from the same population can provide helpful clues in this regard. For example, we have recently reported identification of 35 novel disease genes in the context of developmental delay and intellectual disability in an unselected sample of 337 patients (Anazi et al. 2016). Our identification of an additional 75 novel candidate genes by exome sequencing 348 patients from the same population seems to suggest that the number of yet to be identified genes with mutations etiologic in human disease remains large. We suggest that two of these novel candidates appear to meet the standard cutoff of establishing disease-gene link. Although the phenotype associated with *AKAP6* is nonspecific developmental delay and seizures, the phenotype we observe in the two families with different heterozygous variants in *UBR4* is highly specific and consists of early onset dementia. The remaining candidate genes will require independent confirmation in the future. It is noteworthy that if all 75 novel candidates are confirmed, this will increase the yield of exome sequencing to 83%, which suggests that most clinical exomes that are reported as “negative” by clinical laboratories are reported as such due to interpretation rather than technical limitations as we have proposed in an earlier work (Shamseldin et al. 2016).

In conclusion, this is the largest diagnostic genomic sequencing study of an unselected clinical cohort from Saudi Arabia with suspected genetic disease from a single clinical sequencing lab. In addition to providing numerous examples of phenotypic expansion, we report the first autosomal recessive inheritance of genes that have thus far been linked to human diseases only in a dominant fashion. We add 166 novel variants in known disease genes, and proposed 75 genes as novel disease candidates, two of which are independently mutated in more than one family with the same phenotype. It is hoped that the results of this study will improve the diagnostic yield of genomic sequencing locally and globally.

## Electronic supplementary material

Below is the link to the electronic supplementary material.

Supplementary material 1 (XLSX 132 kb)

## References

[CR1] Abouelhoda M, Faquih T, El-Kalioby M, Alkuraya FS (2016). Revisiting the morbid genome of Mendelian disorders. Genome Biol.

[CR2] Alfares AA, Alfadhel M, Wani T, Alsahli S, Alluhaydan I, Al Mutairi F, Alothaim A, Albalwi M, Alturki S, Al-Twaijri W (2017). A multicenter clinical exome study in unselected cohorts from a consanguineous population of Saudi Arabia demonstrated a high diagnostic yield. Mol Genet Metab.

[CR3] Alkuraya FS (2014). Genetics and genomic medicine in Saudi Arabia. Mol Genet Genom Med.

[CR4] Alkuraya FS (2015). Natural human knockouts and the era of genotype to phenotype. Genome Med.

[CR5] Anazi S, Maddirevula S, Faqeih E, Alsedairy H, Alzahrani F, Shamseldin H, Patel N, Hashem M, Ibrahim N, Abdulwahab F (2016). Clinical genomics expands the morbid genome of intellectual disability and offers a high diagnostic yield. Mol Psychiatry.

[CR6] Bamshad MJ, Ng SB, Bigham AW, Tabor HK, Emond MJ, Nickerson DA, Shendure J (2011). Exome sequencing as a tool for Mendelian disease gene discovery. Nat Rev Genet.

[CR7] Bourassa CV, Meijer IA, Merner ND, Grewal KK, Stefanelli MG, Hodgkinson K, Ives EJ, Pryse-Phillips W, Jog M, Boycott K (2012). VAMP1 mutation causes dominant hereditary spastic ataxia in Newfoundland families. Am J Hum Genet.

[CR8] Boycott KM, Rath A, Chong JX, Hartley T, Alkuraya FS, Baynam G, Brookes AJ, Brudno M, Carracedo A, den Dunnen JT (2017). International cooperation to enable the diagnosis of all rare genetic diseases. Am J Hum Genet.

[CR9] Butcher DT, Cytrynbaum C, Turinsky AL, Siu MT, Inbar-Feigenberg M, Mendoza-Londono R, Chitayat D, Walker S, Machado J, Caluseriu O (2017). CHARGE and Kabuki syndromes: gene-specific DNA methylation signatures identify epigenetic mechanisms linking these clinically overlapping conditions. Am J Hum Genet.

[CR10] Carr IM, Bhaskar S, O’Sullivan J, Aldahmesh MA, Shamseldin HE, Markham AF, Bonthron DT, Black G, Alkuraya FS (2013). Autozygosity mapping with exome sequence data. Hum Mutat.

[CR11] Dheensa S, Shkedi‐Rafid S, Crawford G, Bertier G, Schonstein L, Lucassen A (2016) Management of incidental findings in clinical genomic sequencing studies. eLS 1–7. doi:10.1002/9780470015902.a0025838

[CR12] Grampsas SA, Chandhoke PS, Fan J, Glass MA, Townsend R, Johnson AM, Gabow P (2000). Anatomic and metabolic risk factors for nephrolithiasis in patients with autosomal dominant polycystic kidney disease. Am J Kidney Dis.

[CR13] Group SM (2015). Comprehensive gene panels provide advantages over clinical exome sequencing for Mendelian diseases. Genome Biol.

[CR14] Harrison SM, Riggs ER, Maglott DR, Lee JM, Azzariti DR, Niehaus A, Ramos EM, Martin CL, Landrum MJ, Rehm HL (2016). Using ClinVar as a resource to support variant interpretation. Curr Protoc Hum Genet.

[CR15] Ilgaz Aydinlar E, Rolfs A, Serteser M, Parman Y (2014). Mutation in FAM134B causing hereditary sensory neuropathy with spasticity in a Turkish family. Muscle Nerve.

[CR16] Kalia SS, Adelman K, Bale SJ, Chung WK, Eng C, Evans JP, Herman GE, Hufnagel SB, Klein TE, Korf BR (2016). Recommendations for reporting of secondary findings in clinical exome and genome sequencing 2016 update (ACMG SF v2. 0): a policy statement of the American College of Medical Genetics and Genomics. Genet Med.

[CR17] Kurth I, Pamminger T, Hennings JC, Soehendra D, Huebner AK, Rotthier A, Baets J, Senderek J, Topaloglu H, Farrell SA (2009). Mutations in FAM134B, encoding a newly identified Golgi protein, cause severe sensory and autonomic neuropathy. Nat Genet.

[CR18] Monies D, Maddirevula S, Kurdi W, Alanazy MH, Alkhalidi H, Al-Owain M, Sulaiman RA, Faqeih E, Goljan E, Ibrahim N (2017). Autozygosity reveals recessive mutations and novel mechanisms in dominant genes: implications in variant interpretation. Genet Med.

[CR19] Nimura K, Ura K, Shiratori H, Ikawa M, Okabe M, Schwartz RJ, Kaneda Y (2009). A histone H3 lysine 36 trimethyltransferase links Nk2–5 to Wolf-Hirschhorn syndrome. Nature.

[CR20] Parsons K, Nakatani Y, Nguyen MD (2015). p600/UBR4 in the central nervous system. Cell Mol Life Sci.

[CR21] Patel N, Alkuraya FS (2015). Overlap between CHARGE and Kabuki syndromes: more than an interesting clinical observation?. Am J Med Genet A.

[CR22] Posey JE, Harel T, Liu P, Rosenfeld JA, James RA, Coban Akdemir ZH, Walkiewicz M, Bi W, Xiao R, Ding Y (2017). Resolution of disease phenotypes resulting from multilocus genomic variation. N Engl J Med.

[CR23] Richards S, Aziz N, Bale S, Bick D, Das S, Gastier-Foster J, Grody WW, Hegde M, Lyon E, Spector E (2015). Standards and guidelines for the interpretation of sequence variants: a joint consensus recommendation of the American College of Medical Genetics and Genomics and the Association for Molecular Pathology. Genet Med.

[CR24] Shamseldin HE, Maddirevula S, Faqeih E, Ibrahim N, Hashem M, Shaheen R, Alkuraya FS (2016). Increasing the sensitivity of clinical exome sequencing through improved filtration strategy. Genet Med.

[CR25] Stenson PD, Mort M, Ball EV, Evans K, Hayden M, Heywood S, Hussain M, Phillips AD, Cooper DN (2017). The Human Gene Mutation Database: towards a comprehensive repository of inherited mutation data for medical research, genetic diagnosis and next-generation sequencing studies. Hum Genet.

[CR26] Trujillano D, Bertoli-Avella AM, Kandaswamy KK, Weiss ME, Köster J, Marais A, Paknia O, Schröder R, Garcia-Aznar JM, Werber M (2017). Clinical exome sequencing: results from 2819 samples reflecting 1000 families. Eur J Hum Genet.

[CR27] Yang Y, Muzny DM, Reid JG, Bainbridge MN, Willis A, Ward PA, Braxton A, Beuten J, Xia F, Niu Z (2013). Clinical whole-exome sequencing for the diagnosis of mendelian disorders. N Engl J Med.

[CR28] Yavarna T, Al-Dewik N, Al-Mureikhi M, Ali R, Al-Mesaifri F, Mahmoud L, Shahbeck N, Lakhani S, AlMulla M, Nawaz Z (2015). High diagnostic yield of clinical exome sequencing in Middle Eastern patients with Mendelian disorders. Hum Genet.

